# Evaluation of low doses BPA-induced perturbation of glycemia by toxicogenomics points to a primary role of pancreatic islets and to the mechanism of toxicity

**DOI:** 10.1038/cddis.2015.319

**Published:** 2015-10-29

**Authors:** E Carchia, I Porreca, P J Almeida, F D'Angelo, D Cuomo, M Ceccarelli, M De Felice, M Mallardo, C Ambrosino

**Affiliations:** 1 IRGS, Biogem, Via Camporeale, 83031 Ariano Irpino, Avellino, Italy; 2 STAB VIDA-Investigação e Serviços em Ciências Biológicas, Madan Parque, Caparica, Portugal; 3 Department of Science and Technology, University of Sannio, via Port'Arsa 11, Benevento, Italy; 4 IEOS-CNR, via Pansini 5, Napoli, Italy; 5 Molecular Medicine and Medical Biotechnologies, University of Naples ‘Federico II', Naples, Italy

## Abstract

Epidemiologic and experimental studies have associated changes of blood glucose homeostasis to Bisphenol A (BPA) exposure. We took a toxicogenomic approach to investigate the mechanisms of low-dose (1 × 10^−9 ^M) BPA toxicity in *ex vivo* cultures of primary murine pancreatic islets and hepatocytes. Twenty-nine inhibited genes were identified in islets and none in exposed hepatocytes. Although their expression was slightly altered, their impaired cellular level, as a whole, resulted in specific phenotypic changes. Damage of mitochondrial function and metabolism, as predicted by bioinformatics analyses, was observed: BPA exposure led to a time-dependent decrease in mitochondrial membrane potential, to an increase of ROS cellular levels and, finally, to an induction of apoptosis, attributable to the bigger *Bax*/*Bcl*-2 ratio owing to activation of NF-*κ*B pathway. Our data suggest a multifactorial mechanism for BPA toxicity in pancreatic islets with emphasis to mitochondria dysfunction and NF-*κ*B activation. Finally, we assessed *in vitro* the viability of BPA-treated islets in stressing condition, as exposure to high glucose, evidencing a reduced ability of the exposed islets to respond to further damages. The result was confirmed *in vivo* evaluating the reduction of glycemia in hyperglycemic mice transplanted with control and BPA-treated pancreatic islets. The reported findings identify the pancreatic islet as the main target of BPA toxicity in impairing the glycemia. They suggest that the BPA exposure can weaken the response of the pancreatic islets to damages. The last observation could represent a broader concept whose consideration should lead to the development of experimental plans better reproducing the multiple exposure conditions.

In the past decade, a huge effort has been made to identify xenobiotics that represent a risk factor for glucose homeostasis and diabetes development, impairing hepatocytes function, insulin production/response and, finally, reducing the *β-*cell mass.^1, 2, 3^ Loss of *β-*cells is typically associated to type 1 diabetes (T1D), even though their failure has been involved in the pathogenesis of obesity-associated type 2 diabetes (T2D).^4^ Estrogens and xenoestrogens have a direct role in the regulation of glucose homeostasis, enhancing the secretion of insulin and affecting *β*-cells survival via non-genomic pathway, through estrogen receptor *α* (ER*α*) and *β* (ER*β*) or G protein-coupled estrogen receptor (GPR30).^5, 6, 7, 8^

Bisphenol A (BPA), a well-characterized xenoestrogen, is described as risk factor for development of T2D.^8, 9, 10, 11, 12^ It is used in the manufacture of polycarbonates and epoxy resins. Its polymers, not toxic, are used in food contact materials. Monomers, exerting the estrogenic activity, can seep into the water or food following breakdown of polymers.^13^ The EFSA considers diet as the major source of BPA exposure.^14^ Exposure to BPA is nearly ubiquitous with concentration in human serum ranging from 0.2 to 1.6 ng/ml and it accumulates in fat.^15, 16, 17^ In the last decade, the interest for potential effects of BPA on metabolic disorders is on the rise. Indeed, a positive correlation between diabetes and BPA urinary levels was found combining the data from three epidemiological NHANES studies^18^ while other reports retrieved different degree of associations.^2^ However, increasing *in vitro* and *in vivo* evidences suggest that BPA affects functions of pancreatic islets.^19, 20, 21, 22, 23, 24, 25, 26^ It has been shown that exposure to low doses of BPA, *in vitro*, could cause an increase of insulin content and release in the islets throughout ER pathways^22, 24^ or CREB activation.^19^ Indeed, the *in vivo* exposure to 10 *μ*g/kg/day or 100 *μ*g/kg/day altered the glycemic and insulin curve, favouring insulin resistance.^20^ In murine pancreatic islets, BPA has been shown to act similarly to E_2_ binding membrane-bound ER and exerting non-genomic actions.^26^ In the same context, exposure to low dose of BPA (1 × 10^−9^ M) induced the increase of intracellular [Ca^2+^], insulin release and, finally, the transcription of CREB-dependent genes (e.g., insulin gene).^19^ Furthermore, Lin *et al.*^25^ demonstrated that low concentration of BPA could cause mitochondrial dysfunction and cell death in INS-1E, a rat insulinoma cell line.

The liver also has a relevant role in maintenance of the glucose balance through the regulation of its storage and release. Moreover, this organ is the major site of BPA metabolism^27^ and exposure to BPA has been associated with abnormalities of liver function and hepatic damage.^28, 29, 30^ *In vivo* and *in vitro* studies documented in the hepatocytes an altered gene expression after exposure to different doses of BPA.^31^ An increased apoptosis was also detected in hepatocytes after exposure to reference dose of 50 *μ*g/kg/day, from gestation day 0 to the postnatal day 21 (ref. 32). Perinatal exposure to BPA has been linked to hypermethylation of glucokinase promoter and to inhibition of its gene expression that could contribute to the development of insulin resistance in the adulthood.^33^

The summarized results suggest a clear role of BPA in the impairment of glucose homeostasis. They sketch the mechanisms of BPA toxicity even if the molecular aspects are not completely defined. The use of the ‘omics' approaches has been strongly suggested to explore the mechanisms of toxicity exerted by BPA and other endocrine disrupting chemicals.^34, 35^ Indeed, the gene expression profiles contain a significant amount of information on the current biological conditions leading to a better understanding of related phenotypic and molecular changes.^36^ Therefore, we chose the transcriptomic approach to underscore the key molecular events through which BPA impairs glucose homeostasis in primary cells. Primary *ex vivo* cell cultures have been preferred to immortalized cell lines to preserve different cell types and their interactions within the pancreatic islets and to *in vivo* studies to reduce the number of enrolled animals.

Here we report the results of a study aimed to investigate the mechanisms of toxicity of environmental dose of BPA (1 × 10^−9^ M) on cultured murine primary hepatocytes and pancreatic islets by a toxicogenomic approach. Our work do not identify any significant transcriptional effects in hepatocytes but confirms the pivotal role of mitochondrial dysfunction in the apoptosis induced by BPA exposure of pancreatic islets. We report the map of the slight transcriptome alterations whose summary results in a reduced viability of the pancreatic islets. Importantly, accordingly to our data, BPA exposure impairs the recovery of islet cells from damages, thus suggesting a mechanism for the diabetogen role of BPA.

## Results

### Toxicogenomics of murine primary hepatocytes and pancreatic islets exposed to low dose of BPA

**Figure 1:**
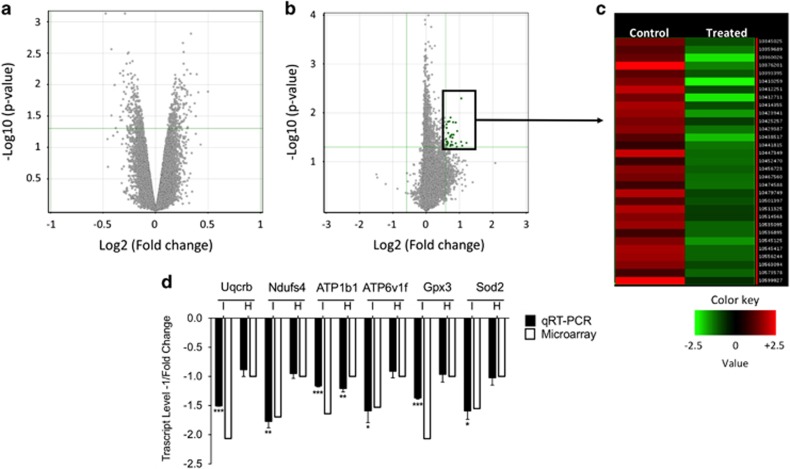
Differentially expressed genes between vehicle and 1x10^−9^ M BPA-treated samples for 48 h. Volcano plots of microarray data of hepatocytes (**a**) and islets (**b**). The y-axis value is the negative logarithm base 10 of the corrected *P*-value. A green horizontal line on the plot represents the user-defined significant threshold for *P-*value. The x-axis is shown as the logarithm base 2 of the fold change in expression level between treated and control. The green lines on the plot represents the user-defined significant threshold for *P-*value (horizontal) and fold change (vertical). Green dots are downregulated genes. (**c**) HeatMap showing the gene expression profile in the pancreatic islets microarray data. The expression value of each gene is mapped to a color-intensity value, as indicated by the color bar; (**d**) qRT-PCR validation of some inhibited genes in treated islets (I) and hepatocytes (H) compared with microarray data. Data are reported as the negative inverse of fold change value. The qRT-PCR results are expressed as the mean±SD of three independent experiments (*N*=3). *P-value <0.05; **P-value <0.01; ***P-value <0.001 compared with vehicle-only-treated cells

**Figure 2:**
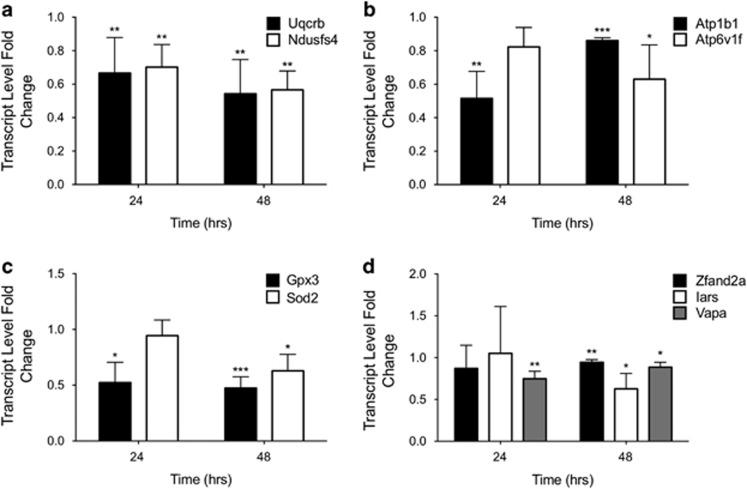
Time-dependent effects of BPA on transcript inhibition. Level of transcripts was determined by qRT-PCR after exposure to 1 × 10^−9^ M BPA for 24 h and 48 h. The selected genes were grouped by functional categories: respiratory chain subunits, *Uqcrb* and *Ndufs4* (**a**); ATP-dependent pump subunits, ATP1b1 and ATP6v1f (**b**); ROS detoxification, Gpx3 and Sod2 (**c**); protein synthesis and degradation, Vapa, Iars, Zfand2a (**d**). Data are reported as fold change values. The results are expressed as the mean±SD of three independent experiments (*N*=3). *P-value <0.05; **P-value <0.01; ***P-value <0.001 compared with vehicle-only-treated islets

To molecularly define the effects that BPA exerted on glucose homeostasis, we analyzed the transcriptional response in two tissues involved in glucose metabolism: liver and pancreas. Cultures of murine primary hepatocytes and pancreatic islets were prepared and exposed to low dose (1 × 10^−9^ M) BPA for 48 h. The time was fixed to analyze the effects on healthy cultured primary cells (see Supplementary Figure S1). Surprisingly, *ex vivo* exposure to 1 × 10^−9^ M BPA did not affect the transcriptome of hepatocytes (Figure 1a). Conversely, a small group of inhibited genes was identified in islets, as shown by the Volcano Plot (Figure 1b) and by the Heatmap (Figure 1c). Twenty-nine genes were differentially expressed. They are listed in Table 1, where the fold changes of microarray and qRT-PCR are reported. Furthermore, for six selected genes, we confirmed the specific inhibition for islets as it was not retrieved in the hepatocytes (Figure 1d). By Ingenuity Pathway Analysis (IPA), some of the inhibited genes were involved in two deregulated Canonical Pathways both pertaining to mitochondrial function: oxidative phosphorylation and mitochondrial dysfunction (Log B-H *P*-value, 2.12). Transcript level of some of these genes was determined to assess the timing of the inhibition. We selected genes encoding components of respiratory chain complexes (*Uqcrb* and *Ndufs4*), proteins involved in cell detoxification (*Gpx3*, *Sod2* and *Zfand2A*), proteins involved in ATP production and/or in insulin exocytosis process (*ATP1b1*, *ATP6v1f* and *Vapa*). Isoleucine-tRNA synthetase (*Iars*) was selected as defects in aminoacylation of tRNA^ILE^ were associated to the impairment of cytochrome c oxidase activity.^37^ To this aim, islets were exposed to 1 × 10^−9^ M BPA for 24 h and 48 h. The data confirmed the inhibition of selected transcripts at 48 h, starting already at 24 h for some of them (Figures 2a and d). The inhibition of *ATP1b1, Vapa* and *Zfand2a* was stronger at 24 h than 48 h (Figures 2b and d). We did not observe any significant modification in the protein levels of some of them (data not shown), maybe owing to the slight reduction of their transcripts with respect to their high cellular content.

### Mitochondrial dysfunction and cell viability in pancreatic islets exposed to low dose of BPA

**Figure 3:**
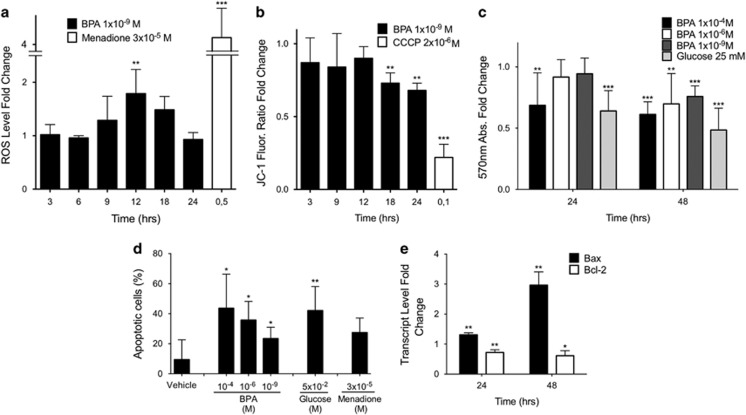
BPA impairs ROS intracellular level, mitochondrial membrane potential and islet viability. (**a**) ROS intracellular level as determined by CellROX reagent and (**b**) measurement of mitochondrial potential as determined by JC-1 reagent. For both assays, equal number of dispersed islets was cultured in the presence of 1x10^−4^ M, 1x10^−9^ M BPA or vehicle only for different times. Menadione 3x10^−5^ M for 30 min (in **a**) and 2x10^−6^ M CCCP (in **b**) were used as positive controls. Fluorescence emission was normalized on protein amount and data are reported as fold change values. (**c**) Murine islets cell viability determined by MTT assay. Dispersed islet cells, in equal number, were cultured in the presence of 1x10^−4^ M, 1x10^−6^ M, 1x10^−9^ M BPA, 25 mM glucose or vehicle only for 24 h or 48 h. Data are reported as fold change values. (**d**) Quantification of apoptotic islet cells by IF-TUNEL staining. Dispersed islet cells were cultured in chamber slides 1x10^−4^ M, 1x10^−6^ M, 1x10^−9^ M BPA, 25 mM glucose, 3x10^−5^ M menadione or vehicle only (DMSO) for 48 h and then visualized under a fluorescence microscope. Data are reported as percentage of TUNEL-positive cells/total cell number (DAPI staining). (**e**) qRT-PCR of Bax and Bcl-2 transcripts in murine pancreatic islets. Level of transcripts was determined by qRT-PCR after exposure to 1x10^−9^ M BPA for 24 h and 48 h. Data are reported as fold change values. All the results are expressed as the mean±SD of three independent experiments (*N*=3). *P-value <0.05; **P-value <0.01; ***P-value <0.001 compared with vehicle-only-treated islets

Starting from the bioinformatic prediction of altered mitochondrial function, we decided to assess the intracellular oxidative stress and mitochondrial activity in the treated islets to confirm functionally the microarray results. The exposure was performed on dispersed pancreatic islet cells, treated with low-dose (1 × 10^−9^ M) BPA for different times. Although we did not find a clear reduction in the protein levels of the deregulated transcripts, we could detect a clear and time-dependent increment of intracellular ROS level after 12 h exposure (fold change 1.79, Figure 3a) and a subsequent impairment of mitochondrial membrane potential at 18 h (0.76) and 24 h (0.74) of treatment (Figure 3b). The BPA effects on islet survival were analyzed by MTT assay. Higher doses (1 × 10^−4^ M and 1 × 10^−6^ M) were also tested. BPA affected the cellular viability of dispersed islets in a dose-dependent manner (Figure 3c). The highest BPA dose was already toxic after 24 h with an activity similar to 25 mM glucose (0.64 and 0.48 at 24 h and 48 h, respectively). Exposure to lower doses of BPA (1 × 10^−6^ M and 1 × 10^−9^ M) reduced the cell viability only at 48 h (0.70 and 0.76, respectively).

The above-reported data suggested that BPA exposure could damage islet cells throughout alteration of mitochondrial function and incrementing ROS cellular level. Both aspects have been implied in the islet viability.^38, 39^ We assessed the apoptosis at single cell level by TUNEL assay performed on dispersed islets exposed for 48 h to high (1 × 10^−4^ M and 1 × 10^−6^ M) and low (1x10^−9^ M) doses of BPA. Glucose (25 mM) and menadione (3 × 10^−5^ M) were also used owing to their ability to induce apoptosis^40^ and ROS generation^41^ in islets. In agreement to the MTT assay, the percentage of apoptotic cells increased with the BPA concentration (Figure 3d and Supplementary Figure S2): the highest BPA dose had similar effects to 25 mM glucose (43.66 and 42.07%, respectively). Noteworthy, the apoptotic effect at 1 × 10^−9^ M BPA was similar to the one exerted by menadione (23.42 and 27.44%, respectively). Molecular aspects were investigated determining the cellular level of two transcripts involved in the apoptotic process: the pro-apoptotic *Bax* and anti-apoptotic *Bcl-2*. As reported in Figure 3e, *Bax* transcript level significantly increased at any analyzed time point. Indeed, it was induced about three times after 48 h exposure. Conversely, the expression of the anti-apoptotic gene *Bcl-2* was inhibited by exposure to 1 × 10^−9^ M BPA after 24 h (fold change 0.67) and not deeply modified at longer time.

### Cellular pathways involved in pancreatic islet damage following low-dose BPA exposure

**Figure 4:**
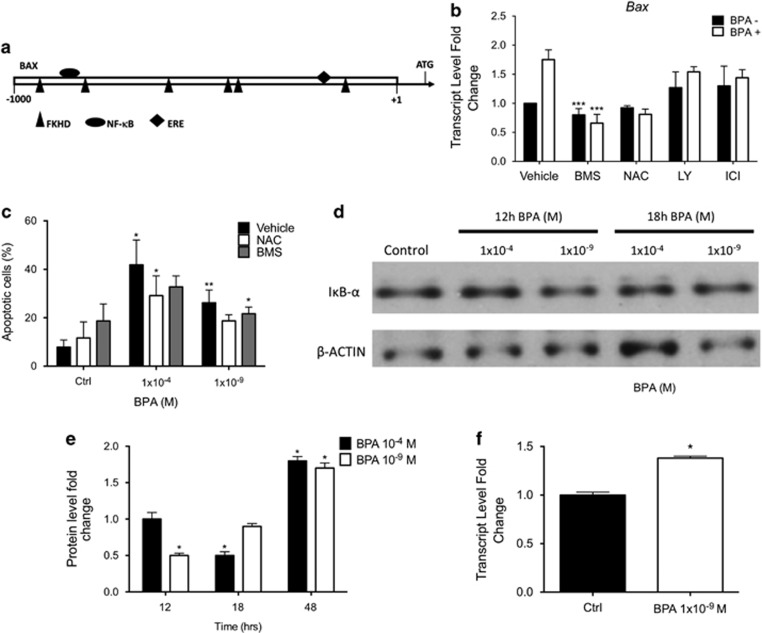
Pathways involved in BPA apoptosis triggering. (**a**) Schematic representation of transcription binding sites for fork head domain factors (FKHD), nuclear factor kappa B (NF-*κ*B) and estrogen responsive elements (ERE) predicted by Genomatix software in Bax gene promoter fragment −1000/+50 bp; (**b**) qRT-PCR analyses of Bax transcript in murine pancreatic islets cultured in the presence or absence of 1 × 10^−9^ M BPA, 3 mM NAC, 2 × 10^−5^ M BMS-345541, 1 × 10^−5^ M LY-294002 or 1 × 10^−5^ M ICI-182,780, for 48 h. Data are reported as fold change values. *P-value <0.05; **P-value <0.01; ***P-value <0.001 compared with BPA-only-treated islets; (**c**) IF-TUNEL staining of cells co-exposed to BPA and ROS and NF-*κ*B inhibitor. Dispersed islet cells were cultured in chamber slides in the presence or absence of 1 × 10^−4^ M BPA, 1 × 10^−9 ^M BPA, 3 mM NAC, 2 × 10^−5^ M BMS-345541 for 48 h and then visualized under a fluorescence microscope. Data are reported as percentage of TUNEL-positive cells/total cell number (DAPI staining). *P-value <0.05; **P-value <0.01; ***P-value <0.001 compared with BPA-only-treated islets; (**d**) IkB-*α* protein was determined in whole cellular extracts prepared from intact murine pancreatic islets cultured in the presence or absence of 1 × 10^−4^ M and 1 × 10^−9^ M BPA for 12 h and 18 h by western blot; (**e**) protein quantification of IkB-*α* in murine pancreatic islets cultured as already reported (**d** and Supplementary Figure S3). The signal intensity was determined with imageJ software and normalized on *β*-actin protein signal intensity. Data are reported as fold change values. *P-value <0.05; **P-value <0.01; ***P-value <0.001 compared with vehicle-only-treated islets; (**f**) qRT-PCR quantification of IkB-*α* transcript in murine pancreatic islets. Level of transcripts was determined by qRT-PCR after exposure to 1 × 10^−9^ M BPA for 12 h. Data are reported as fold change values. *P-value <0.05; **P-value <0.01; ***P-value <0.001 compared with vehicle-only-treated islets. All the results are expressed as the mean±SD of three independent experiments (*N*=3)

Although BPA best-known targets are ERs, BPA is able to exert its activity through other signaling pathways, as NF-*κ*B.^42^ The IPA analyses predicted several BPA targets as Forkhead Domain Factors (FOXO, *P*-value 0.02) and NF-*κ*B (*P*-value 0.04), among the others. We verified through a bioinformatics tool, Genomatix, if ER, NF-*κ*B and FOXO binding sites were predicted in the *Bax* promoter (Figure 4a) as well as in the promoters of the other previously selected genes. They were found in all the analyzed promoters (data not shown). Given the role of *Bax* in the apoptosis, we focused on this gene and assessed the effects, on BPA activity, of inhibitors of the cited pathways: BMS-345541 (NF-*κ*B inhibitor), LY-294002 (PI3K/AKT inhibitor, a regulator of FOXO) and ICI-182780 (*ERα* antagonist). The role of NAC (a ROS inhibitor) was also assessed. To this aim, purified islets were co-exposed for 48 h to 1 × 10^−9^ M BPA and different inhibitors. The co-exposure of the islets to BPA and ICI-182780 or LY-294002 did not significantly affect the upregulation of *Bax* (Figure 4b). Even though NAC and BMS-345541 exposure, slightly and not significantly, inhibited *Bax* transcript, both inhibitors deeply affected the *Bax* mRNA increase due to BPA. This molecular result was phenotypically confirmed assessing the ability of NAC and BMS-345541 to revert the BPA-dependent apoptosis in treated cells. To this aim, TUNEL assay was performed on dispersed islets co-exposed for 48 h to BPA (1x10^−4^ M and 1x10^−9^ M) and NAC or BMS-345541. As reported in Figure 4c, both inhibitors significantly reduced the number of apoptotic cells, in co-treated cells, when compared with BPA-only-treated cells (1x10^−4^ M BPA: Vehicle 41.8%, NAC 29.2%, BMS 32.7% 1 × 10^−9^M BPA: Vehicle 26.2%, NAC 18.7%, BMS 21.6%), pointing to a clear role of ROS induction and NF-*κ*B activation in the process. The activation of the NF-*κ*B pathway by low dose BPA exposure was further confirmed assessing the cellular level of IkB*-α* that has to be degraded to allow NF-*κ*B nuclear translocation. To this aim, whole cellular lysates were prepared from pancreatic islets treated with BPA (1 × 10^−4^ M and 1 × 10^−9^ M) and IkB-*α* cellular content assayed by western blotting. The time points, 12 h, 18 h and 48 h, were chosen according to ROS level, mitochondrial potential and gene expression assays, respectively. As shown in Figure 4d, IkB-*α* protein level was decreased after 12 h of exposure at 1x10^−9^ M BPA, in correspondence with the peak of ROS production. At later time points, 18 h (Figure 4d) and 48 h (Figure 4e and Supplementary Figure S3), it increased, probably as a result of the NF-*κ*B activation. Indeed, *IkB-α* is a well-known NF-*κ*B target gene^43^ and the increase in its transcript is detectable by qRT-PCR already at 12 h of exposure (Figure 4f). At 1x10^−4^ M BPA exposure, we observed a degradation of IkB-*α* at a later time (18 h), maybe due to more stressing condition or an inverse dose-effect response already described for BPA. The NF-*κ*B activity increase was further documented by assessing nuclear p65/Rel A in the nuclei of BPA-treated islet cells by whole-mount immunohistochemistry (Supplementary Figure S4).

### Low dose exposed murine pancreatic islets show an impaired response to glucose stress *in vitro* and *in vivo*

**Figure 5:**
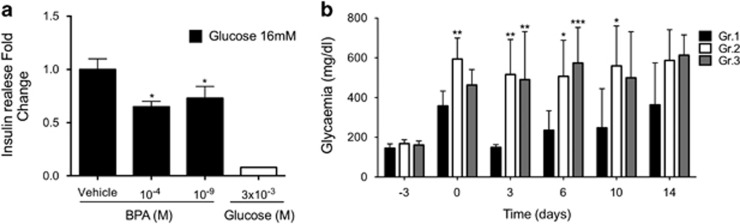
Effects of BPA on islets functionality. (**a**) Insulin secretion after 1 h of 16 mM glucose stimulation of islets exposed to 1 × 10^−4^ M, or 1 × 10^−9^ M BPA. Data are reported as fold change values. The results are expressed as the mean±SD of three independent experiments (*N*=3). *P-value <0.05; **P-value <0.01; ***P-value <0.001 compared with vehicle-only-treated islets; (**b**) glycemia measurements in hyperglycemic mice transplanted with pancreatic islets. The animals were intravenously injected (day −3) with STZ for hyperglycemia induction and transplanted with pancreatic islets from healthy syngenic donors, three days later (day 0). Transplanted islets were cultured for 48 h with (Gr.2, *N*=5 and Gr.3, *N*=4) or without (control group, Gr.1, *N*=5) 1 × 10^−9^ M BPA. Gr.2 animals were watered with 50 *μ*g/kg bw/day BPA from the day of STZ injection until the day of killing. *P-value <0.05; **P-value <0.01; ***P-value <0.001 compared with control animals (Gr.1)

The described results suggested that BPA toxicity on murine *ex vivo* cultured pancreatic islets was exerted altering the mitochondria functionality and lowering the cellular viability. As no massive effects were detected on cell viability, we speculated that BPA could act, mainly, altering the islet response/recovery from an injury, as glucose overload. To test this hypothesis, we assessed the ATP production in islet cells, dispersed and cultured, co-exposed to BPA (1 × 10^−9^ M) and glucose (25 mM). As reported in Table 2, the cells co-treated with glucose and 1 × 10^−6^ M or 1x10^−9^ M BPA showed a reduction of ATP content up to 0.42 and 0.35 fold change versus the glucose-only-treated islets. Furthermore, the treatment with both 1 × 10^−4^ M and 1 × 10^−9^ M BPA for 48 h reduced the secretion of insulin in response to 1 h stimulation with 16 mM glucose (fold change 0.65 and 0.73, respectively; Figure 5a). We confirmed the results testing the ability of the treated islets to restore the glycemia after their transplantation in diabetic mice. For *in vivo* experiment, primary islets were prepared and cultured for 48 h with 1 × 10^−9^ M BPA or vehicle alone. To ensure the restoration of the normal glycemia level despite the apoptosis induced by BPA treatment, 400 BPA-treated and control islets were transplanted in STZ injected mice.^44^ As shown in Figure 5b, the transplant with BPA pre-treated islets was unable to restore normal glycemic level neither in BPA treated (Gr.2) nor in normal water (Gr.3)-administered mice at any time. We also found a loss of weight in the BPA-treated animals significantly greater in treated mice than controls (data not shown). As already shown,^45^ the BPA exposure worsened the effects of STZ injection suggesting that the BPA exposure affected islet activity in injuring conditions.

## Discussion

Epidemiological and experimental studies showed that exposure to BPA elicited alteration in pancreatic islets function and glucose homeostasis. In this work, we studied the effects of BPA on murine primary hepatocytes and pancreatic islets through a toxicogenomic approach, mimicking the worldwide population exposure to BPA low-dose (1 × 10^−9^ M).^15, 17^ No transcriptional effect was retrieved in primary hepatocytes, even though some effects have been described in other experimental settings,^46^ whereas in islets only a few transcripts were inhibited. They were mainly involved in the deregulation of mitochondrial activity and, despite the slight effects detected at mRNA level, the summary of their impairments functionally resulted in the alteration of ROS cellular content and mitochondrial membrane potential.

The toxicogenomic analysis allowed us to have a snapshot of the global status of the islets, indicating the mitochondrion as the key organelle affected by BPA exposure. Mitochondria are critical for the maintenance of *β*-cell function coupling glucose stimulus to insulin release^47, 48^ and are targets of BPA toxicity.^32, 49^ Moreover, alterations in mitochondrial dynamics have been reported among the mechanisms underlying the development of T2D and obesity.^50^ Indeed, recent studies indicated that BPA exposure could cause profound mitochondrial structural defects and decrease of mitochondrial activity in pancreatic islets.^23^ Here, we report that BPA inhibits the expression of genes involved in mitochondrial activity leading to impairment of the organelle function and its damage. This results in a reduction of insulin secretion following glucose stimulation and in an increase in the apoptosis. Both mechanisms of BPA toxicity are suggested by the toxicogenomic analyses and could contribute to the impairment of blood glucose homeostasis. Other authors report demonstrated that BPA treatment induces an increment in the insulin cellular content *in vivo* and *in vitro* after 4 days and 48 h of treatment, respectively^20, 24^ and an increment of insulin release *in vitro* after 1 h of stimulation.^22^ Our results suggest that longer exposure to BPA (48 h) reduces insulin secretion in response to 16 mM glucose. Considering our result, we suggest that the increment of the intracellular insulin content observed by others in the 48 h treated islets is possible owing to the impairment in the secretion mechanism that results in the intracellular accumulation of the protein. Noteworthy, the decrease of the *Vapa* transcript, codifying for a SNARE protein, here reported could be a part of the mechanism behind this alteration of insulin content/release.

BPA is a xenoestrogen acting through nuclear ERs and membrane-bound ERs.^8^ Despite that, the reported bioinformatics analyses predicted the deregulation of other transcription factors, as NF-*κ*B and FOXO families. Therefore, we experimentally confirmed the activation of the NF-*κ*B pathway (Figure 4) and ruled out a clear involvement of the FOXO proteins and, above all, ER*α* at the analyzed time points. Indeed, previous studies have suggested a direct involvement of ER*α*, ER*β* and GPR30 in mediating low-dose BPA-increased insulin release or protein level. The ER*α*-dependent increase in the insulin transcript was reported only at shorter exposure time, whereas the protein induction at longer time (48 h) using ER*α*−/− and ER*β*−/− animal.^24^ If it is undoubted that ERs have a pivotal role also in insulin release, it must be noted that this effect has been reported at shorter time point^22^ and that the activation of other signaling pathways involved in the process has been shown. Therefore, it is not surprising to us that other transcriptional regulators can mediate the reported effects. Although we cannot rule out a role of ERs, as we have specifically inhibited only ER*α*, we do identify NF-*κ*B pathway as a target of BPA activity in pancreatic islets in our experimental setting. Furthermore, in immortalized thyrocytes,^42^ we have already described that ERs are not playing a direct role in mediating BPA transcriptional effects using a specific reporter system underlining meanwhile the role of NF-*κ*B pathway. In light of the reported results, BPA acts in the same way in islet cells activating NF-*κ*B pathway after inducing intracellular ROS level, (Figure 3). These results are in agreement with the findings that BPA exposure increase ROS cellular amount^51^ resulting in the activation of the NF-*κ*B pathway in an IKK-dependent manner,^52^ here confirmed by the reduction in IkB*-α* cellular content in treated pancreatic islets.

**Figure 6:**
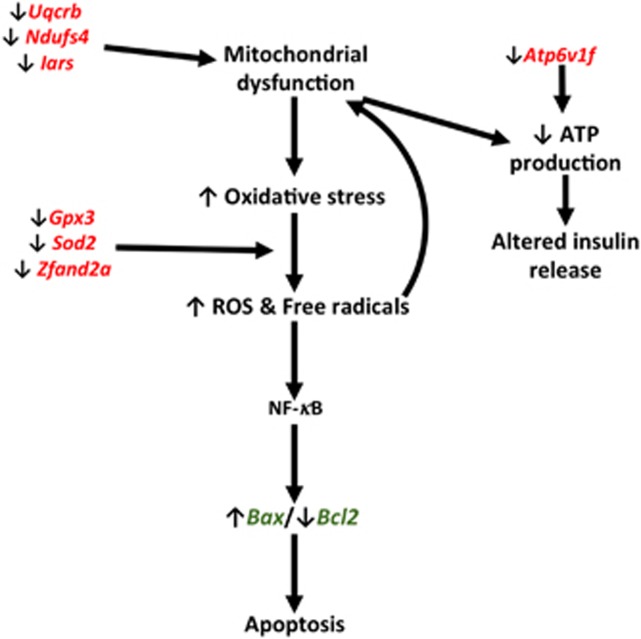
Mechanisms of BPA mitochondrial dysfunction and apoptosis in pancreatic islets. Genes deregulated upon BPA exposure as determined by microarray analysis (in red) and qRT-PCR (in green)

Indeed, the inhibited transcripts suggest different mechanisms through which BPA could determine islet failure and death, depicted in Figure 6. The downregulation of transcripts involved in mitochondrial respiratory chain (*Uqcrb* and *Ndufs4* Figure 2a), as well as *ATP6v1f* (Figure 2b), points to an impaired oxidative phosphorylation. This causes defects in glucose-stimulated ATP production, therefore, directly impairing the insulin secretion. Noteworthy, *ATP1b1,* a subunit of sodium/potassium-transporting ATPase, is also inhibited in our experiments (Figure 2b) suggesting that alterations in the Ca^2+^ and K^+^ homeostasis could be also involved in islet failure (Figure 6).^50^ Furthermore, the inhibited expression of respiratory chain genes could promote the leakage of electrons inducing free radical accumulation to which the inhibition of the ROS scavenging transcripts (*Sod2* and *Gpx3*, Figure 2c) could contribute,^53^ resulting in the NF-*κ*B activation.^52^ In summary, BPA could exert toxic activity throughout two complementary mechanisms centered on mitochondria: enhancement of the oxidative stress and drop of the ROS scavenging systems, promoting the apoptosis. Their simultaneous impairment amplifies the effects of BPA. Despite the weakness of the detected effects, in terms of gene transcript inhibition, they determine a reduced response of the islets to further injuries, as the exposure to high glucose *in vitro* and *in vivo*. Indeed, our findings evidence that the BPA-induced damage cannot be recovered, even after dropping the exposure. In agreement with recent results, where the treatment was performed with a 100 times higher BPA dose,^45^ we show that BPA exacerbates the diabetogenic effects of STZ. Furthermore, our data suggest that BPA pre-treated islets are impaired and apoptotic, therefore, not efficiently engrafting when transplanted in hyperglycemic mice, even if not longer exposed.

Many evidences show the role of environmental pollutants in diabetes development. Indeed, there were 285 million people worldwide with diabetes in 2010 and this number is estimated to increase by 54% within 2030,^54^ indicating that factors, other than genetic, could equally contribute. Their clear identification is important to explain the epidemic diffusion of diabetes and to develop preventive measures. To this aim, our findings are significant as they characterize, at transcriptomic level, the reported impairment of islet activity and viability. The toxicogenomic approach reveals different mechanisms through which BPA exerts its effects on pancreatic islets. The future assessment of the real risks related to the BPA exposure needs to turn the attention on the interplay among genetic and multiple environmental factors to better match the real world.

## Materials and Methods

### Animals

C57/B6 male mice, 22–27 gr., 5–8 weeks old, were obtained from Biogem s.c.a.r.l. (Ariano Irpino, Italy). Mice were housed in polysulfone cages with *ad libitum* access to water and normal diet under specific pathogen-free facility at 22 °C±2° temperature and 55%±15% relative humidity with 12 h light: 12 h darkness cycle and 18±2 changes of air per hour. All animal experimentation aspects respected the regulations and guidelines of Italy and the European Union and the NIH Principles of Laboratory Animal Care (NIH, publication no. 85-23, revised 1985) and have been evaluated and approved by the ethics committee ‘Comitato Etico per la Sperimentazione Animale' (CESA) of IRGS, Biogem.

### Murine pancreatic islets

Primary cultures of pancreatic islets were prepared by pancreas bile duct perfusion, Collagenase P digestion (0.8 mg/ml, Roche GmbH, Mannheim, Germany) and cultured as already described.^55^ Briefly, islets were purified from exocrine tissue by differential centrifugation on Histopaque 1077 (Sigma-Aldrich, St. Louis, MO, USA) and HBSS (w/o Ca and Mg, Sigma-Aldrich) density gradient. The islet yield and quality was checked on an inverted microscope. The islets were handpicked and cultured at 37 °C with 5% CO_2_. Islets were cultured in RPMI 1640 (Sigma-Aldrich)+10% fetal bovine serum (FBS, Sigma-Aldrich)+1% Pen/Strep (Sigma-Aldrich). A total 250–300 islets per 6 cm dish with 3 ml of media were used, avoiding stressing condition. The islets were treated with 1 × 10^−4^ M, 1 × 10^−6^ M or 1 × 10^−9^ M BPA, 25 mM glucose, 3x10^−5^ M menadione, 3 mM NAC, 2 × 10^−5^ M BMS-345541, 1x10^−5^ M LY-294002, 1x10^−5^ M ICI-182780 or vehicle (DMSO). All the chemicals were purchased from Sigma-Aldrich. Treatments were performed for time points ranging from 3 h to 48 h; at longer time fibroblast proliferation and islets senescence was detected (Supplementary Figure S1). When required, islets were dispersed as previously described.^56^ For all the experiments, to minimize the effects of mice interindividual variations, the islets from three or more pancreas were pooled and then re-divided for treatments.

### Murine primary hepatocytes

Primary culture of murine hepatocytes were obtained by liver collagenase perfusion and digestion as previously described.^57^ Briefly, after anesthesia and laparotomy, a PE10 catheter was introduced in the portal vein. The liver was perfused with a pre-digestion solution (HBSS w/o Mg and Ca+0.5 mM EGTA+25 mM Hepes+1% Pen/Strep (Sigma-Aldrich), pH 7.4) pre-warmed to 37 °C for 10 min at the rate of 5 ml/min, afterwards perfused with pre-warmed digestion solution (William's E Medium+15 mM Hepes+1% Pen/Strep+0.32 mg/ml Collagenase type IV (Sigma-Aldrich), pH 7.4) at the same rate for 10 min. The digested liver was excised and hepatocytes were released by gently shaking into 15 ml of hepatocyte isolation medium (William's E+4% FBS+1% Pen/Strep (Sigma-Aldrich)). To access the quality of the perfusion, trypan blue staining assessed the cell viability. Then, the cells were plated in collagen type I (Sigma-Aldrich)-coated dishes with hepatocyte isolation medium. After 6 h at 37 °C, the cells were attached and the medium was replaced with hepatocyte growth medium (William's E+4% heat-inactivated FBS+1% Pen/Strep+50 ng/ml Epidermal Growth Factor+1 mg/ml insulin+10 mg/ml Transferrin+1.3 mg/ml Hydrocortisone (Sigma-Aldrich)). The treatments were conducted as described above. Treatments longer than 48 h were not conducted as *in vitro* mesenchymal transition was observed (Supplementary Figure S1).

### MTT and ATP assay

MTT (Sigma-Aldrich) and ATPlite assay (PerkinElmer Life Sciences, Inc, Zaventem, Belgium) were performed on dispersed islets, grown in the appropriate plates, according to manufacturer's instructions. For MTT, absorbance at 570 nm was read in EnVision 2103 Multilabel Reader (PerkinElmer Life Sciences, Inc). For ATP assay, luminescence was measured in Orion II microplate luminometer (Berthold Detection Systems GmbH, Pforzheim, Germany). The luminescence level was normalized on protein amount.

### RNA extraction and qRT-PCR

RNA was extracted from isolated islets or hepatocytes using Trizol reagent (Life Technologies Italia, Monza, Italy) and quantified with the NanoDrop spectrophotometer ND-1000. For qRT-PCR, 1 *μ*g of total RNA was reverse-transcribed (QuantiTect Reverse Transcription Kit, Qiagen), according to the manufacturer's instructions. qRT-PCR experiments were conducted using Life Technologies Italia 7300 Real-Time PCR System and Power SYBR Green Master Mix (Life Technologies Italia). The subsequent analysis was performed following Hellemans *et al.*^58^ models for qRT-PCR relative quantification and inter-run calibration with proper error. Data were normalized on the relative expression of three reference genes (Gapdh, Tubulin and *β*-2 microglobulin).^58^ Primers were designed using NCBI Primer Blast tool (http://www.ncbi.nlm.nih.gov/tools/primer-blast). Their sequences are reported below:

*ATP1b1*, 5′-CTTCCGTCCTAATGACCCCA-3′ (forward) and 5′-TGATTGATGTCGCCCCGTTC-3′ (reverse); *ATP6v1f* 5′-ATCGAAGACACTTTCAGGCAA-3′ (forward) and 5′-ATGCTCCTTGGACGGGATCT-3′ (reverse); *Bax*, 5′-ACAGATCATGAAGACAGGGG-3′ (forward) and 5′-CAAAGTAGAAGAGGGCAACC-3′ (reverse); *Bcl-2*, 5′-ATGACTGAGTACCTGAACCGGCAT-3′ (forward) and 5′-GGGCCATATAGTTCACAAAGGCA-3′ (reverse); *Gpx3*, 5′-AAACAGGAGCCAGGCGAGAACT-3′ (forward) and 5′-CCCGTTCACATCTCCTTTCTCAAA-3′ (reverse); *Iars*, 5′-GACTTGGAGGAGGTAGTGTGC-3′ (forward) and 5′-GATGGGATGGTCAGGTGGTC-3′ (reverse); *IkBα*, 5′-GCACTTGGCAATCATCCACG-3′ (forward) and 5′-CAAGTGGAGTGGAGTCTGCTG-3′ (reverse); *Ndufs4,* 5′-TGGCTACAGCTGCCGTTTCCG-3′ (forward) and 5′-GGTCAGCGGTTGATGCCCAA -3′ (reverse); *Sod2,* 5′-CGTGAACAATCTCAACGCCACCGA-3′ (forward) and 5′-CCTCCAGCAACTCTCCTTTGGGTT-3′ (reverse); *Uqcrb*, 5′-GCGGGCCGATCTGCTGTTTC-3′ (forward) and 5′- GCCTCATAGTCAGGTCCAGGGCT-3′ (reverse); *Vapa*, 5′- GAGATGTGTGTTTGAAATGCCGA-3′ (forward) and 5′- GGTCCGTCTTGTTTGGATGC-3′ (reverse); *Zfand2a* 5′-ACCCGTGAGTGCCAGGTGAT-3′ (forward) and 5′- AACAGTGCTTCCCCAAGTCAGGA-3′ (reverse); *B2M* 5′-CCGAACATACTGAACTGCTA-3′ (forward) and 5′-TGCTATTTCTTTCTGCGTGC-3′ (reverse); *Gapdh*, 5- ACCACAGTCCATGCCATCAC-3′ (forward) and 5′- CACCACCCTGTTGCTGTAGCC-3′ (reverse); *Tub*, 5′- CAACACCTTCTTCAGTGAGACAGG-3′ (forward) and 5′-TACATGATCTCCTTGCCAATGGT-3′ (reverse).

### Microarray

Gene expression profiling experiments and data analyses were conducted as elsewhere described.^59^ The Affimetrix platform was used. cRNA was generated by using the Affymetrix One-Cycle Target Labeling and Control Reagent kit (Affymetrix Inc., Foster City, CA, USA), according to the manufacturer's instructions, starting from 5 *μ*g of total RNA. Biotinylated cRNA was hybridized to the Mouse MOE 430 2.0 Genome Arrays (Affymetrix Inc.). Chips were washed and scanned on the Affymetrix Complete GeneChip System, generating digitized image data (DAT) files. The data sets obtained were analyzed with GeneSpring GX 12 Software (Agilent Technologies Inc., Santa Clara, CA, USA). Robust multichip average (RMA) algorithm^60^ was used for summarization and normalization. Hybridization quality was assessed by spiked-in controls. Principal Component Analysis (PCA) was performed to check data quality that resulted adequate for all the samples. Transcripts were filtered by their signal intensity values, selecting transcripts with intensity values between 20 and 100 percentile in at least one out of each set samples for differential analysis. Differentially expressed transcripts between exposed samples *versus* controls were filtered for absolute fold change ⩾1.5 and corrected *P*-value ⩽0.05. Statistical analysis was performed using one-way ANOVA adjusted for multiple comparison by the Benjamini–Hochberg method.

Functional annotation for differentially expressed transcripts was performed using Ingenuity Pathway Analysis (IPA; http://www.ingenuity.com), a web-based tool for the identification of biological functions as well as canonical pathways that are most significant to the data set. Fisher Exact test was used to calculate the *P*-value determining the likelihood that the association between the set of focus genes in the data set and a given process or pathway is due to chance alone. Corrected *P*-value calculation (based on the Benjamini–Hochberg method) controlled the error rate in analysis results and focus in on the most significant biological functions associated with deregulated genes.

### Evaluation of cellular ROS production

CellROX green reagent assay (Life Technologies Italia), to measure intracellular ROS amount, was performed on dispersed islets, grown in the appropriate plates, according to manufacturer's instructions. Fluorescence was measured with EnSpire Multimode Plate Reader (ex./em. 485/520 nm, PerkinElmer Life Sciences, Inc) after treatment with BPA 1 × 10^−9^ M different times Treatment with 3 × 10^−5^ M menadione was used as positive control. The fluorescence level was normalized on protein amount.

### Mitochondrial membrane potential measurements

Mitoprobe JC-1 assay kit (Life Technologies Italia) was used to measure mitochondrial membrane potential. The assay was performed on dispersed islets, grown in the appropriate plates, according to manufacturer's instructions. Fluorescence was measured with EnSpire Multimode Plate Reader (ex./em. 514/530, 575/590 nm, PerkinElmer Life Sciences, Inc) after treatment with BPA 1 × 10^−9^ M at different times. Treatment with 2x10^−6^ M carbonyl cyanide 3-chlorophenylhydrazone (CCCP) for 5 min was used as positive control. The fluorescence level was normalized on protein amount. A decrease in the red/green fluorescence intensity ratio was taken as indicator of mitochondrial depolarization.

### Western blot

Islets were lysed in buffer containing 50 mM HEPES, pH 7.5, 150 mM NaCl, 1 mM MgCl_2_, 1 mM CaCl_2_, 4 mM EDTA, 1% Triton X-100, 10% glycerol, 50 mM NaF and 10 mM sodium pyrophosphate, supplemented with 0.1 mM phenylmethylsul- fonyl fluoride, 5 ng/ml leupeptin, 1 μg/ml aprotinin and 2 mM Na_3_VO_4_ for 1 h at 4 °C. After centrifugation (14 000 g for 30 min) supernatants were collected and protein concentration measured by Bradford assay. Equal protein amount (20 *μ*g) from the islets lysates were separated by SDS-PAGE. Membranes were probed with primary antibody against IkB*-α* (sc-371, Santa Cruz Biotechnology Inc., Dallas, TX, USA). Mouse *β*-actin antibody (Sigma-Aldrich) was used to normalize protein levels. Band intensities were quantified by ImageJ software (1.48v, National Health Institute, NIH, Bethesda, MD, USA).

### TUNEL assay and islet immunofluorescence

Apoptotic cells were visualized with TUNEL assay kit (*In Situ* Cell Death Kit, Fluorescein, Roche GmbH), according to manufacturer's instructions, counterstained with a 1 *μ*g/ml DAPI solution and visualized under fluorescence microscope (Carl Zeiss, Oberkochen, Germany). After treatment with 1 × 10^−4^ M, 1 × 10^−6^ M, 1 × 10^−9^ M BPA, glucose 25 mM or 3 × 10^−5^ M menadione or vehicle only (24 h and 48 h), the cells were fixed (4% paraformaldehyde-PBS) and permeabilized (0.1% Triton X-100-PBS). For immunofluorescence, whole islets were grown in the presence of 1 × 10^−9^ M BPA or vehicle only. The NF-*κ*B p65 staining was conducted with the rabbit mAb (Ab7970, Abcam plc, Cambridge, UK), according to manufacturer's instruction. Rodhamine conjugated secondary antibody (711-025-752, Jackson ImmunoResearch Laboratories Inc., West Grove, PA, USA) was used. After counterstaining with 1 *μ*g/ml DAPI solution, the islets were visualized under fluorescence microscope. Quantitative comparison of NF-*κ*B p65 sublocalization was determined using ImageJ. Cell nuclei were determined drawing a region of interest (ROI) on merged images. Defined ROIs were copied on 570 nm images and measured. A minimum of 20 nuclei/sample were analyzed from three independent experiments.

### Insulin secretion assay

Insulin secretion assay was conducted as elsewhere described.^24^ Briefly, 10 purified islets were handpicked accordingly to a homogeneous size and incubated for 48 h with appropriate treatment. At the end of the treatment, islets were washed in buffer solution (20 mM NaCl, 5 mM KCl, 25 mM NaHCO_3_, 1.1 mM MgCl_2_ and 2.5 mM CaCl_2_, 3 mM glucose, pH 7.35) for 2 h, when gassed with 95% O_2_ and 5% CO_2_. After that, islets were incubated in the same buffer with 3 or 16 mM glucose. After 1 h, the medium was collected and insulin measured by ELISA immunoassay kit (EZRMI-13 k, EMD Millipore, Billerica, MA, USA).

### *In vivo* study

To induce hyperglycemia, mice were intravenous injected with a single dose of Streptozotocin (STZ) dissolved in PBS at 150 mg/kg bw and divided into three groups: control group (Gr.1), Gr.2 and Gr.3. Three days after STZ injection, the glycemia was measured (Glucocard MX blood glucose meter, A. Menarini Diagnostics s.r.l, Florence, Italy) and only animals with glycemia levels ⩾250 mg/dl were chosen to continue the study. A graft of 400 pancreatic islets coming from syngenic healthy mice was transplanted under the kidney capsule of each mouse. Gr.2 and Gr.3 animals received islets incubated for 48 h with 1 × 10^−9^ M BPA. Gr.1 animals received islets incubated with vehicle only. Gr.2 animals were administered with 50 *μ*g/kg/day BPA (dissolved in water at a concentration 8.77 × 10^−9^ M) from the day of STZ injection for the duration of the study. Gr.1 and Gr.3 were administered with normal water. Glycemia and body weight were measured bi-weekly until killing at day 15 post transplant.

### Statistical and bioinformatics analyses

The statistical analyses have been performed with Student's *t*-test, unless otherwise indicated. In all the cases, probability *P*-values below 0.05 were considered significant. *, **, *** indicate *P*-value <0.05, <0.01, <0.001, respectively. Data from at least three independent experiments were considered for the statistical analysis. Unless otherwise indicated, data are reported as fold change values calculated as ratio between average results in treated and control samples. The results are expressed as the mean±SD of three independent experiments. Hypothetical transcriptional factors binding sites in the gene promoter were identified loading their sequence ranging from −1000/+50 bp in Genomatix suite software (Genomatix Software GmbH, http://www.genomatix.de), choosing a relative profile score of 80%.^61^

## Figures and Tables

**Table 1:** Genes downregulated in murine pancreatic islets exposed to 1x10^−9^ M BPA for 48 h as determined by microarray and qRT-PCR

**Transcript cluster ID**	**Refseq accession number**	**Gene symbol**	**Gene description**	**Fold change**	
				**Microarray**	**qRT-PCR**
10410259	NM_026219	*Uqcrb*	Ubiquinol-cytochrome c reductase binding protein	−2.3	−1.51
10376201	NM_001083929	*Gpx3*	Glutathione peroxidase 3	−2.07	−1.37
10423941	NM_025736	*Ttc35*	Tetratricopeptide repeat domain 35	−1.91	−2.63
10438517	NM_145939	*Alg3*	Asparagine-linked glycosylation 3 homolog	−1.88	−1.54
10545125	NM_022891	*Rpl23*	Ribosomal protein L23	−1.84	—
10447349	NM_019936	*Cript*	Cysteine-rich PDZ-binding protein	−1.84	—
10429387	NM_023587	*Ptplb*	Protein tyrosine phosphatase-like	−1.76	—
10545417	NM_145569	*Mat2a*	Methionine adenosyltransferase II, alpha	−1.75	—
10535095	NM_133349	*Zfand2a*	Zinc finger, AN1-type domain 2°	−1.73	−1.10
10556244	NR_033336	*Snora23*	Small nucleolar RNA, H/ACA box 23	−1.72	—
10412251	NM_010887	*Ndufs4*	NADH dehydrogenase (ubiquinone) Fe-S protein 4	−1.69	−1.77
10467560	NM_133352	*Tm9sf3*	Transmembrane 9 superfamily member 3	−1.69	
10456723	—	*BC031181*	—	−1.67	
10414355	NM_178684	*Mapk1ip1l*	Mapk 1 interacting protein 1-like	−1.66	
10599927	NM_008032	*Aff2*	AF4/FMR2 family, member 2	−1.66	
10479749	NM_001013376	*Rpp38*	Ribonuclease P/MRP 38 subunit (human)	−1.65	—
10359689	NM_009721	*Atp1b1*	ATPase, Na+/K+ transporting, beta 1 polypeptide	−1.64	−1.16
10511325	NM_026147	*Rps20*	Ribosomal protein S20	−1.62	—
10474588	—	*2900064A13Rik*	RIKEN cDNA 2900064A13 gene	−1.58	—
10563094	NM_013725	*Rps11*	Ribosomal protein S11	−1.57	—
10345025	NM_172015	*Iars*	Isoleucine-tRNA synthetase	−1.56	−1.60
10441815	NM_013671	*Sod2*	Superoxide dismutase 2, mitochondrial	−1.55	−1.59
10393395	NM_011358	*MRF-1*	Splicing factor, arginine/serine-rich 2 (SC-35)	−1.55	—
10452470	NM_013933	*Vapa*	VAMP, associated protein A	−1.54	−1.13
10573578	—	*BC056474*	—	−1.54	—
10536895	NM_025381	*Atp6v1f*	ATPase, H+ transporting, lysosomal V1 subunit F	−1.53	−1.59
10514568	NM_053157	*Tm2d1*	TM2 domain containing 1	−1.53	—
10501397	NM_026198	*Tmem167b*	Transmembrane protein 167B	−1.52	—
10425257	NM_027231	*Polr2f*	Polymerase (RNA) II (DNA directed) polyp. F	−1.51	—

**Table 2:** BPA affects ATP content in pancreatic islets cultured in the presence of 25 mM glucose

**BPA**	**Glucose 25 mmol/l**	**Normalized on vehicle-treated cells**			**Normalized on glucose-treated cells**		
		**Relative luminescence level fold change**	**St. Dev.**	***P*****-value**	**Relative luminescence level fold change**	**St. Dev.**	***P*****-value**
Vehicle	−	1.000	0.247				
10^−6^ M	−	0.983	0.330	0.922			
10^−9^ M	−	1.047	0.244	0.745			
−	+	0.261	0.314	0.001	1.000	0.474	
10^−6^ M	+	0.088	0.107	0.0001	0.418	0.177	0.018*
10^−9^ M	+	0.102	0.103	0.0001	0.348	0.137	0.009**

Data are reported as fold change values calculated as ratio between average luminescence/total protein content in treated and control cells. *P-value <0.05; **P-value <0.01; compared with glucose-only-treated islets

## Abbreviations

BPA: bisphenol A
CCCP: carbonyl cyanide 3-chlorophenylhydrazone
CREB: cAMP response element-binding protein
E_2_: 17*β*-estradiol
EDTA: ethylenediaminetetraacetic acid
EGTA: ethylene glycol tetraacetic acid
ER: estrogen receptor
FBS: fetal bovine serum
FOXO: forkhead domain factors
GPR30: G protein-coupled estrogen receptor
HBSS: Hank's Balanced Salt Solution
IkB: NF-kappa-B inhibitor alpha
IPA: Ingenuity Pathway Analysis
NAC: *N*-acetylcysteine
NF-*κ*B: nuclear factor kappa-light-chain-enhancer of activated B cells
NHANES: National Health and Nutrition Examination Survey
PBS: phosphate-buffered saline
ROI: region of interest
ROS: reacting oxygen species
STZ: streptozoticin
T1D: diabetes type 1
T2D: diabetes type 2

## References

[bib1] Kirkley AG, Sargis RM. Environmental endocrine disruption of energy metabolism and cardiovascular risk. Curr Diab Rep 2014; 14: 494. 24756343 10.1007/s11892-014-0494-0 PMC4067479

[bib2] Chevalier N, Fénichel P. Endocrine disruptors: new players in the pathophysiology of type 2 diabetes? Diabetes Metab 2014; 41: 107–115. 25454091 10.1016/j.diabet.2014.09.005

[bib3] Peng H, Hagopian W. Environmental factors in the development of Type 1 diabetes. Rev Endocr Metab Disord 2006; 7: 149–162. 17203405 10.1007/s11154-006-9024-y

[bib4] Cnop M, Welsh N, Jonas JC, Jörns A, Lenzen S, Eizirik DL et al. Mechanisms of pancreatic -cell death in type 1 and type 2 diabetes: many differences, few similarities. Diabetes 2005; 54: S97–S107. 16306347 10.2337/diabetes.54.suppl_2.s97

[bib5] Nadal A, Alonso-Magdalena P, Soriano S, Quesada I, Ropero AB. The pancreatic beta-cell as a target of estrogens and xenoestrogens: Implications for blood glucose homeostasis and diabetes. Mol Cell Endocrinol 2009; 304: 63–68. 19433249 10.1016/j.mce.2009.02.016

[bib6] Ropero AB, Alonso-Magdalena P, Quesada I, Nadal A. The role of estrogen receptors in the control of energy and glucose homeostasis. Steroids 2008; 73: 874–879. 18249429 10.1016/j.steroids.2007.12.018

[bib7] Liu S, Le May C, Wong WP, Ward RD, Clegg DJ, Marcelli M et al. Importance of extranuclear estrogen receptor-alpha and membrane G protein-coupled estrogen receptor in pancreatic islet survival. Diabetes 2009; 58: 2292–2302. 19587358 10.2337/db09-0257 PMC2750222

[bib8] Alonso-Magdalena P, Ropero AB, Soriano S, Quesada I, Nadal A. Bisphenol-A: a new diabetogenic factor? Hormones (Athens) 2010; 9: 118–126. 20687395 10.1007/BF03401277

[bib9] Ropero AB, Alonso-Magdalena P, García-García E, Ripoll C, Fuentes E, Nadal A. Bisphenol-A disruption of the endocrine pancreas and blood glucose homeostasis. Int J Androl 2008; 31: 194–200. 17971160 10.1111/j.1365-2605.2007.00832.x

[bib10] Mirmira P, Evans-Molina C. Bisphenol A, obesity, and type 2 diabetes mellitus: genuine concern or unnecessary preoccupation? Transl Res 2014; 164: 13–21. 24686036 10.1016/j.trsl.2014.03.003 PMC4058392

[bib11] Fenichel P, Chevalier N, Brucker-Davis F. Bisphenol A: an endocrine and metabolic disruptor. Ann Endocrinol (Paris) 2013; 74: 211–220. 23796010 10.1016/j.ando.2013.04.002

[bib12] Andra SS, Kalyvas H, Andrianou XD, Charisiadis P, Christophi CA, Makris KC. Preliminary evidence of the association between monochlorinated bisphenol A exposure and type II diabetes mellitus: a pilot study. J Environ Sci Health A Tox Hazard Subst Environ Eng 2015; 50: 243–259. 25594118 10.1080/10934529.2015.981111

[bib13] EFSA CEF Panel. Scientific Opinion on the risks to public health related to the presence of bisphenol A (BPA) in foodstuffs. Part I–Exposure assessment. EFSA J 2015; 13: 3978.

[bib14] EFSA CEF Panel. Scientific Opinion on the risks to public health related to the presence of bisphenol A (BPA) in foodstuffs. PART II–Toxicological assessment and risk characterisation. EFSA J 2015; 13: 3978.

[bib15] Vandenberg LN, Hauser R, Marcus M, Olea N, Welshons WV. Human exposure to bisphenol A (BPA). Reprod Toxicol 2007; 24: 139–177. 17825522 10.1016/j.reprotox.2007.07.010

[bib16] Fernandez MF, Arrebola JP, Taoufiki J, Navalón A, Ballesteros O, Pulgar R et al. Bisphenol-A and chlorinated derivatives in adipose tissue of women. Reprod Toxicol 2007; 24: 259–264. 17689919 10.1016/j.reprotox.2007.06.007

[bib17] Calafat AM, Ye X, Wong L-Y, Reidy JA, Needham LL. Exposure of the U.S. population to bisphenol A and 4-tertiary-octylphenol: 2003-2004. Environ Health Perspect 2008; 116: 39–44. 18197297 10.1289/ehp.10753 PMC2199288

[bib18] Shankar A, Teppala S. Relationship between urinary bisphenol A levels and diabetes mellitus. J Clin Endocrinol Metab 2011; 96: 3822–3826. 21956417 10.1210/jc.2011-1682 PMC3232607

[bib19] Quesada I, Fuentes E, Viso-León MC, Soria B, Ripoll C, Nadal A. Low doses of the endocrine disruptor bisphenol-A and the native hormone 17beta-estradiol rapidly activate transcription factor CREB. FASEB J 2002; 16: 1671–1673. 12207000 10.1096/fj.02-0313fje

[bib20] Alonso-Magdalena P, Morimoto S, Ripoll C, Fuentes E, Nadal A. The estrogenic effect of bisphenol A disrupts pancreatic beta-cell function *in vivo* and induces insulin resistance. Environ Health Perspect 2006; 114: 106–112. 10.1289/ehp.8451 PMC1332664 16393666

[bib21] Alonso-Magdalena P, Vieira E, Soriano S, Menes L, Burks D, Quesada I et al. Bisphenol A exposure during pregnancy disrupts glucose homeostasis in mothers and adult male offspring. Environ Health Perspect 2010; 118: 1243–1250. 20488778 10.1289/ehp.1001993 PMC2944084

[bib22] Soriano S, Alonso-Magdalena P, García-Arévalo M, Novials A, Muhammed SJ, Salehi A et al. Rapid insulinotropic action of low doses of bisphenol-A on mouse and human islets of Langerhans: role of estrogen receptor β. PLoS One 2012; 7: e31109. 22347437 10.1371/journal.pone.0031109 PMC3275611

[bib23] Song L, Xia W, Zhou Z, Li Y, Lin Y, Wei J et al. Low-level phenolic estrogen pollutants impair islet morphology and β-cell function in isolated rat islets. J Endocrinol 2012; 215: 303–311. 22946080 10.1530/JOE-12-0219

[bib24] Alonso-Magdalena P, Ropero AB, Carrera MP, Cederroth CR, Baquié M, Gauthier BR et al. Pancreatic insulin content regulation by the estrogen receptor ER alpha. PLoS One 2008; 3: e2069. 18446233 10.1371/journal.pone.0002069 PMC2323613

[bib25] Lin Y, Sun X, Qiu L, Wei J, Huang Q, Fang C et al. Exposure to bisphenol A induces dysfunction of insulin secretion and apoptosis through the damage of mitochondria in rat insulinoma (INS-1) cells. Cell Death Dis 2013; 4: e460. 23328667 10.1038/cddis.2012.206 PMC3563994

[bib26] Alonso-Magdalena P, Ropero AB, Soriano S, García-Arévalo M, Ripoll C, Fuentes E et al. Bisphenol-A acts as a potent estrogen via non-classical estrogen triggered pathways. Mol Cell Endocrinol 2012; 355: 201–207. 22227557 10.1016/j.mce.2011.12.012

[bib27] Pottenger LH, Domoradzki JY, Markham DA, Hansen SC, Cagen SZ, Waechter JM Jr. The relative bioavailability and metabolism of bisphenol A in rats is dependent upon the route of administration. Toxicol Sci 2000; 54: 3–18. 10746927 10.1093/toxsci/54.1.3

[bib28] Bindhumol V, Chitra KC, Mathur PP. Bisphenol A induces reactive oxygen species generation in the liver of male rats. Toxicology 2003; 188: 117–124. 12767684 10.1016/s0300-483x(03)00056-8

[bib29] Hanioka N, Jinno H, Nishimura T, Ando M. Suppression of male-specific cytochrome P450 isoforms by bisphenol A in rat liver. Arch Toxicol 1998; 72: 387–394. 9708877 10.1007/s002040050518

[bib30] Rönn M, Kullberg J, Karlsson H, Berglund J, Malmberg F, Orberg J et al. Bisphenol A exposure increases liver fat in juvenile fructose-fed Fischer 344 rats. Toxicology 2013; 303: 125–132. 23142792 10.1016/j.tox.2012.09.013

[bib31] Marmugi A, Ducheix S, Lasserre F, Polizzi A, Paris A, Priymenko N et al. Low doses of bisphenol A induce gene expression related to lipid synthesis and trigger triglyceride accumulation in adult mouse liver. Hepatology 2012; 55: 395–407. 21932408 10.1002/hep.24685

[bib32] Xia W, Jiang Y, Li Y, Wan Y, Liu J, Ma Y et al. Early-life exposure to bisphenol a induces liver injury in rats involvement of mitochondria-mediated apoptosis. PLoS One 2014; 9: e90443. 24587367 10.1371/journal.pone.0090443 PMC3938763

[bib33] Ma Y, Xia W, Wang DQ, Wan YJ, Xu B, Chen X et al. Hepatic DNA methylation modifications in early development of rats resulting from perinatal BPA exposure contribute to insulin resistance in adulthood. Diabetologia 2013; 56: 2059–2067. 23748860 10.1007/s00125-013-2944-7

[bib34] Harding AK, Daston GP, Boyd GR, Lucier GW, Safe SH, Stewart J et al. Endocrine disrupting chemicals research program of the U.S. Environmental Protection Agency: summary of a peer-review report. Environ Health Perspect 2006; 114: 1276–1282. 16882539 10.1289/ehp.8875 PMC1552001

[bib35] Hamadeh HK. Prediction of Compound Signature Using High Density Gene Expression Profiling. Toxicol Sci 2002; 67: 232–240. 12011482 10.1093/toxsci/67.2.232

[bib36] Kiyosawa N, Manabe S, Yamoto T, Sanbuissho A. Practical application of toxicogenomics for profiling toxicant-induced biological perturbations. Int J Mol Sci 2010; 11: 3397–3412. 20957103 10.3390/ijms11093397 PMC2956103

[bib37] Degoul F, Brulé H, Cepanec C, Helm M, Marsac C, Leroux J et al. Isoleucylation properties of native human mitochondrial tRNAIle and tRNAIle transcripts. Implications for cardiomyopathy-related point mutations (4269, 4317) in the tRNAIle gene. Hum Mol Genet 1998; 7: 347–354. 9466989 10.1093/hmg/7.3.347

[bib38] Kanak MA, Takita M, Kunnathodi F, Lawrence MC, Levy MF, Naziruddin B. Inflammatory response in islet transplantation. Int J Endocrinol 2014; 2014: 451035. 24883060 10.1155/2014/451035 PMC4021753

[bib39] Thomas DA, Stauffer C, Zhao K, Yang H, Sharma VK, Szeto HH et al. Mitochondrial targeting with antioxidant peptide SS-31 prevents mitochondrial depolarization, reduces islet cell apoptosis, increases islet cell yield, and improves posttransplantation function. J Am Soc Nephrol 2007; 18: 213–222. 17151329 10.1681/ASN.2006080825

[bib40] Federici M, Hribal M, Perego L, Ranalli M, Caradonna Z, Perego C et al. High glucose causes apoptosis in cultured human pancreatic islets of Langerhans: a potential role for regulation of specific Bcl family genes toward an apoptotic cell death program. Diabetes 2001; 50: 1290–1301. 11375329 10.2337/diabetes.50.6.1290

[bib41] Broniowska KA, Mathews CE, Corbett JA. Do β-cells generate peroxynitrite in response to cytokine treatment? J Biol Chem 2013; 288: 36567–36578. 24194521 10.1074/jbc.M113.522243 PMC3868769

[bib42] Gentilcore D, Porreca I, Rizzo F, Ganbaatar E, Carchia E, Mallardo M et al. Bisphenol A interferes with thyroid specific gene expression. Toxicology 2013; 304: 21–31. 23238275 10.1016/j.tox.2012.12.001

[bib43] Sun SC, Ganchi PA, Ballard DW, Greene WC. NF-kappa B controls expression of inhibitor I kappa B alpha: evidence for an inducible autoregulatory pathway. Science 1993; 259: 1912–1915. 8096091 10.1126/science.8096091

[bib44] Ohzato H, Porter J, Monaco AP, Montana E, Maki T. Minimum number of islets required to maintain euglycemia and their reduced immunogenicity after transplantation into diabetic mice. Transplantation 1993; 56: 270–274. 8356579 10.1097/00007890-199308000-00003

[bib45] Kang HS, Yang H, Ahn C, Kang HY, Hong EJ, Jaung EB. Effects of xenoestrogens on streptozotocin-induced diabetic mice. J Physiol Pharmacol 2014; 65: 273–282. 24781736

[bib46] Huc L, Lemarié A, Guéraud F, Héliès-Toussaint C. Low concentrations of bisphenol A induce lipid accumulation mediated by the production of reactive oxygen species in the mitochondria of HepG2 cells. Toxicol In Vitro 2012; 26: 709–717. 22515966 10.1016/j.tiv.2012.03.017

[bib47] Supale S, Li N, Brun T, Maechler P. Mitochondrial dysfunction in pancreatic β cells. Trends Endocrinol Metab 2012; 23: 477–487. 22766318 10.1016/j.tem.2012.06.002

[bib48] Maechler P, Kennedy ED, Pozzan T, Wollheim CB. Mitochondrial activation directly triggers the exocytosis of insulin in permeabilized pancreatic beta-cells. EMBO J 1997; 16: 3833–3841. 9233793 10.1093/emboj/16.13.3833 PMC1170007

[bib49] Wang P, Luo C, Li Q, Chen S, Hu Y. Mitochondrion-mediated apoptosis is involved in reproductive damage caused by BPA in male rats. Environ Toxicol Pharmacol 2014; 38: 1025–1033. 25461564 10.1016/j.etap.2014.10.018

[bib50] Santulli G, Pagano G, Sardu C, Xie W, Reiken S, D'Ascia SL et al. Calcium release channel RyR2 regulates insulin release and glucose homeostasis. J Clin Invest 2015; 125: 1968–1978. 25844899 10.1172/JCI79273 PMC4463204

[bib51] Kovacic P. How safe is bisphenol A? Fundamentals of toxicity: metabolism, electron transfer and oxidative stress. Med Hypotheses 2010; 75: 1–4. 20371154 10.1016/j.mehy.2010.03.002

[bib52] Gloire G, Legrand-Poels S, Piette J. NF-kappaB activation by reactive oxygen species: fifteen years later. Biochem Pharmacol 2006; 72: 1493–1505. 16723122 10.1016/j.bcp.2006.04.011

[bib53] Graciano MFR, Valle MMR, Kowluru A, Curi R, Carpinelli AR. Regulation of insulin secretion and reactive oxygen species production by free fatty acids in pancreatic islets. Islets 2011; 3: 213–223. 21750413 10.4161/isl.3.5.15935

[bib54] Shaw JE, Sicree RA, Zimmet PZ. Global estimates of the prevalence of diabetes for 2010 and 2030. Diabetes Res Clin Pract 2010; 87: 4–14. 19896746 10.1016/j.diabres.2009.10.007

[bib55] Natalicchio A, Labarbuta R, Tortosa F, Biondi G, Marrano N, Peschechera A et al. Exendin-4 protects pancreatic beta cells from palmitate-induced apoptosis by interfering with GPR40 and the MKK4/7 stress kinase signalling pathway. Diabetologia 2013; 56: 2456–2466. 23995397 10.1007/s00125-013-3028-4

[bib56] Chowdhury A, Dyachok O, Tengholm A, Sandler S, Bergsten P. Functional differences between aggregated and dispersed insulin-producing cells. Diabetologia 2013; 56: 1557–1568. 23604550 10.1007/s00125-013-2903-3 PMC3671110

[bib57] Li W-C, Ralphs KL, Tosh D. Isolation and culture of adult mouse hepatocytes. Methods Mol Biol 2010; 633: 185–196. 20204628 10.1007/978-1-59745-019-5_13

[bib58] Hellemans J, Mortier G, De Paepe A, Speleman F, Vandesompele J. qBase relative quantification framework and software for management and automated analysis of real-time quantitative PCR data. Genome Biol 2007; 8: R19. 17291332 10.1186/gb-2007-8-2-r19 PMC1852402

[bib59] Porreca I, D'Angelo F, Gentilcore D, Carchia E, Amoresano A, Affuso A et al. Cross-species toxicogenomic analyses and phenotypic anchoring in response to groundwater low-level pollution. BMC Genomics 2014; 15: 1067. 25475078 10.1186/1471-2164-15-1067 PMC4301944

[bib60] Irizarry RA, Bolstad BM, Collin F, Cope LM, Hobbs B, Speed TP. Summaries of Affymetrix Gene Chip probe level data. Nucleic Acids Res 2003; 31: e15. 12582260 10.1093/nar/gng015 PMC150247

[bib61] Cartharius K, Frech K, Grote K, Klocke B, Haltmeier M, Klingenhoff A et al. MatInspector and beyond: promoter analysis based on transcription factor binding sites. Bioinformatics 2005; 21: 2933–2942. 15860560 10.1093/bioinformatics/bti473

