# Supplementary figures and images for: Evaluation of low doses BPA-induced perturbation of glycemia by toxicogenomics points to a primary role of pancreatic islets and to the mechanism of toxicity
Source: Cell Death Dis. 2015 Oct 29;6(10):e1959–. doi: 10.1038/cddis.2015.319 (PMC5399181; doi:10.1038/cddis.2015.319)

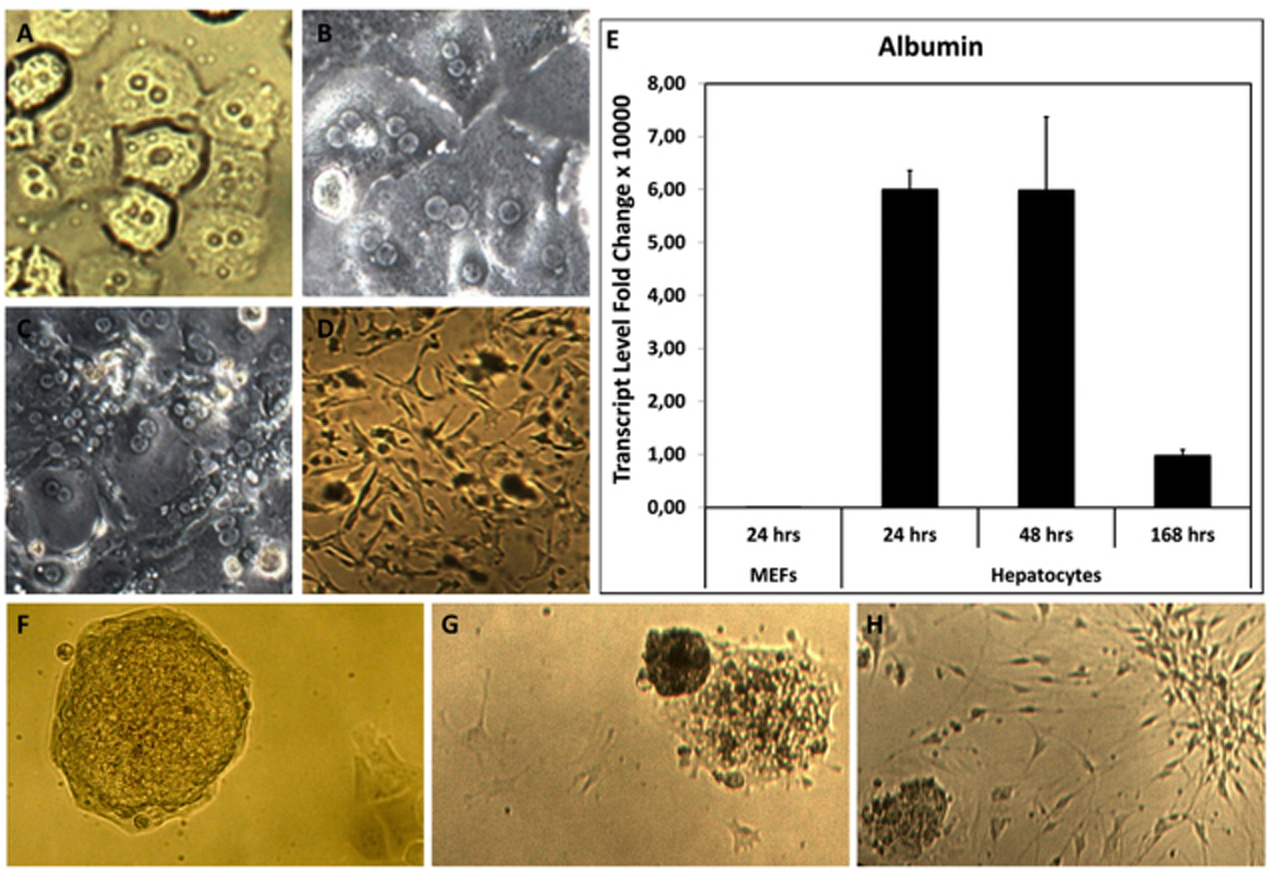

Supplement: Supplementary Figure S1 [file cddis2015319x1.tif]

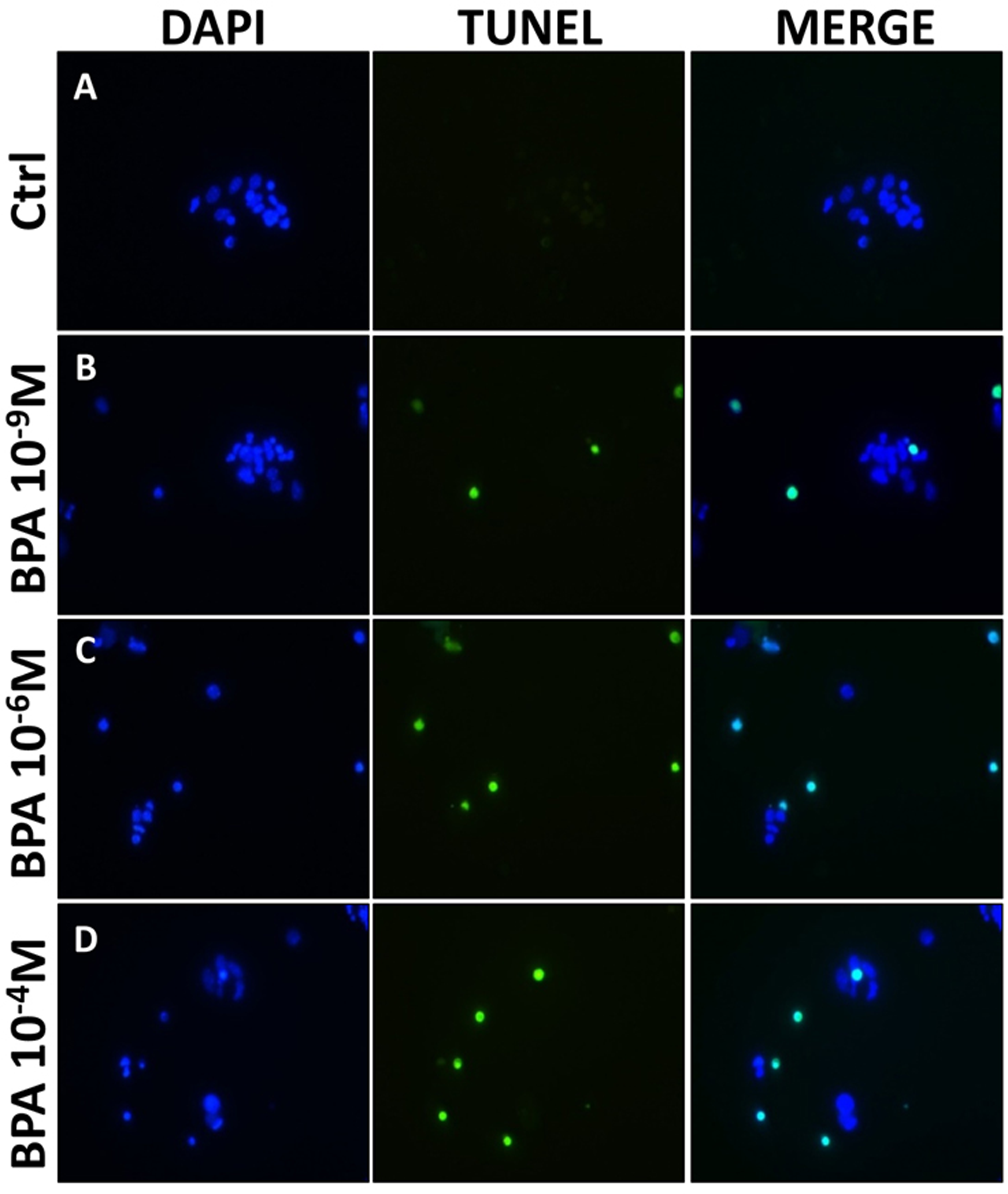

Supplement: Supplementary Figure S2 [file cddis2015319x2.tif]

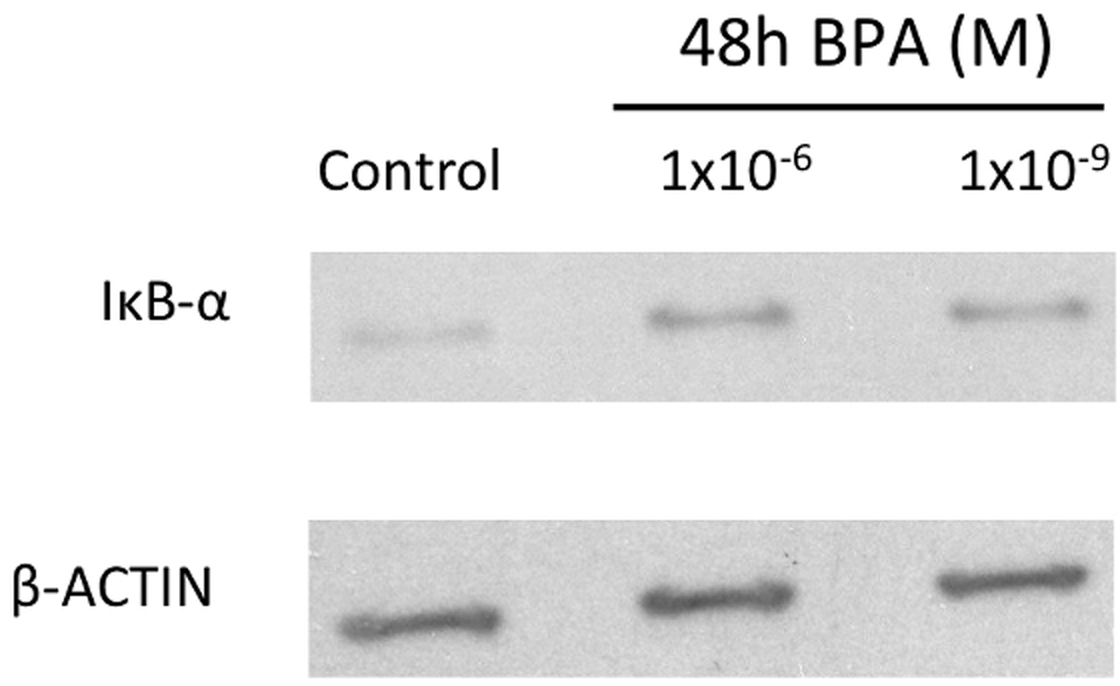

Supplement: Supplementary Figure S3 [file cddis2015319x3.tif]

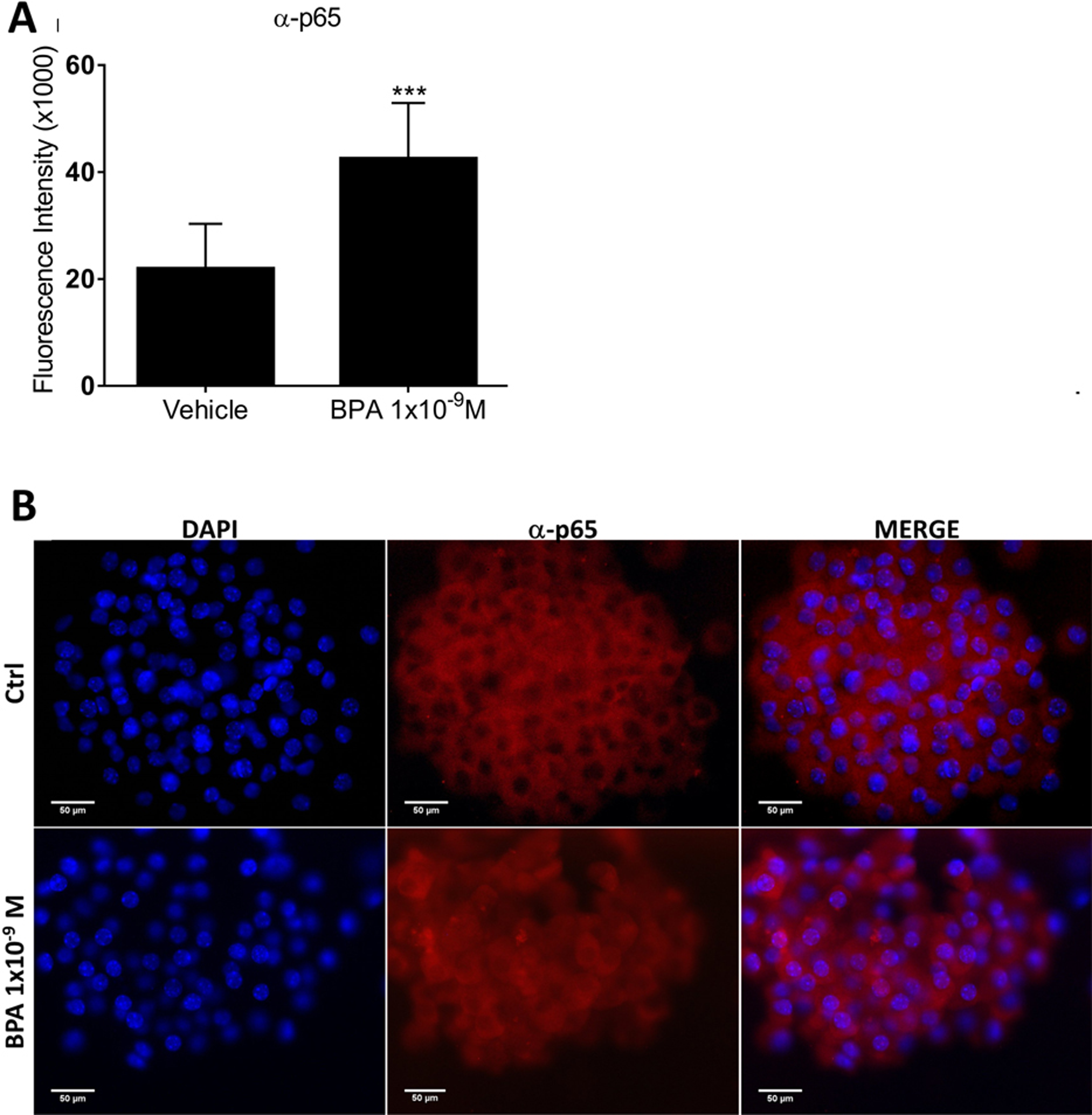

Supplement: Supplementary Figure S4 [file cddis2015319x4.tif]
