# Supplementary material for: Evaluation of low doses BPA-induced perturbation of glycemia by toxicogenomics points to a primary role of pancreatic islets and to the mechanism of toxicity
Source: Cell Death Dis. 2015 Oct 29;6(10):e1959–. doi: 10.1038/cddis.2015.319 (PMC5399181; doi:10.1038/cddis.2015.319)
Supplement: Supplementary Figure Legends [file cddis2015319x5.doc]

***Fig. S1*** *Pancreatic islets senescence and hepatocytes mesenchymal transition evaluation. (****A, B, C, D):*** *Primary culture of murine liver cells. After 12h of culture, parenchymal hepatocytes are attached to the dish’s surface, and showed typical hexagonal cobblestone-like morphology with one or two round nuclei (A, 20X magnification). After 24h, they spread on the dish’s surface, establish intercellular contacts and maintain their morphology (B, 20X). After 48h, they begin to assume a tapered shape but rounded nuclei are still visible (C, 10X). Parenchymal hepatocytes lost their epithelial cell morphology within 7 days in culture, and transformed into more flattened fibroblast-like cells (D, 10X). (****E):*** *Albumin gene expression quantification as hepatocyte molecular marker by qRT-PCR. The data are reported as ratio between average expression of albumin gene in MEFs and in diverse time cultured hepatocytes****. (F, G, H):*** *Primary culture of murine pancreatic islets. After 12h of culture, islets present a healthy aspect with clearly defined border and compact structure (F, 20x magnification). After 96h, islets expel the central necrotic core, loose their compactness and defined border (G, 20x magnification). After 7 days, there is a fibroblast proliferation in plate (H, 10x magnification).*

***Fig. S2*** *Pancreatic islet cells apoptosis induced by treatment with diverse concentration of BPA for 48h as determined by IF-TUNEL assay. Representative images of apoptotic cells stained with DAPI, to evidence nuclei, TUNEL reagent, to notice apoptosis and merged stains at 40x magnification. Islet cells were exposed to: vehicle only (DMSO)* ***(A)****, 1x10^-9^ M* ***(B)****, 1x10^-6^ M* ***(C)****, 1x10^-4^ M* ***(D)*** *BPA.*

***FIG. S3*** *IkBα protein determination by western blot after 48h treatment with 1x10^-9^ and 1x10^-6^ M BPA. Beta-actin was used to normalise protein level.*

***FIG. S4*** *.* ***(A):*** *Fluorescence intensity quantification of NF-κB p65 signal in nuclei of stained pancreatic islet. The signal intensity was determined with ImageJ software on vehicle only and 1x10^-9^M 48h BPA treated islets. The results are expressed as the mean ± standard deviation of diverse field analyzed of three independent experiments (N=3). ***p-value<0.001 compared with vehicle only treated islets.* ***(B):*** *Representative immunofluorescent images of pancreatic islets treated with vehicle only (DMSO, upper, 63x) or 1x10^-9^M BPA (lower, 63x) co-stained with anti-p65 Ab (red) and DAPI for nuclei identification (blue).*
